# Educational and Training Interventions Aimed at Healthcare Workers in the Detection and Management of People With Mental Health Conditions in South and South-East Asia: A Systematic Review

**DOI:** 10.3389/fpsyt.2021.741328

**Published:** 2021-10-11

**Authors:** Kamrun Nahar Koly, Cleo Baskin, Ivylata Khanam, Mala Rao, Sabrina Rasheed, Graham R. Law, Farhana Sarker, Shamini Gnani

**Affiliations:** ^1^Health System and Population Studies Division, International Centre for Diarrhoeal Disease Research, Bangladesh (icddr, b), Dhaka, Bangladesh; ^2^Department of Primary Care and Public Health, Imperial College London, London, United Kingdom; ^3^School of Health and Social Care, University of Lincoln, Lincoln, United Kingdom; ^4^South London and Maudsley NHS Foundation Trust, London, United Kingdom

**Keywords:** health system, mental health, education and training, capacity building, healthcare workers, interventions

## Abstract

**Background:** To bridge significant mental health treatment gaps, it is essential that the healthcare workforce is able to detect and manage mental health conditions. We aim to synthesise evidence of effective educational and training interventions aimed at healthcare workers to increase their ability to detect and manage mental health conditions in South and South-East Asia.

**Methods:** Systematic review of six electronic academic databases from January 2000 to August 2020 was performed. All primary research studies were eligible if conducted among healthcare workers in South and South-East Asia and reported education and training interventions to improve detection and management of mental health conditions. Quality of studies were assessed using Modified Cochrane Collaboration, ROBINS-I, and Mixed Methods Appraisal Tools and data synthesised by narrative synthesis. Results are reported according to Preferred Reporting Items for Systematic Reviews and Meta-analysis guidelines. A review protocol was registered with the PROSPERO database (CRD42020203955).

**Findings:** We included 48 of 3,654 screened articles. Thirty-six reported improvements in knowledge and skills in the detection and management of mental health conditions. Training was predominantly delivered to community and primary care health workers to identify and manage common mental health disorders. Commonly used training included the World Health Organization's mhGAP guidelines (*n* = 9) and Cognitive Behavioural Therapy (*n* = 8) and were successfully tailored and delivered to healthcare workers. Digitally delivered training was found to be acceptable and effective. Only one study analysed cost effectiveness. Few targeted severe mental illnesses and upskilling mental health specialists or offered long-term follow-up or supervision. We found 21 studies were appraised as low/moderate and 19 as high/critical risk of bias.

**Interpretation:** In low resource country settings, upskilling and capacity building of primary care and community healthcare workers can lead to better detection and management of people with mental health disorders and help reduce the treatment gap.

**Systematic Review Registration:** https://www.crd.york.ac.uk/prospero/, identifier CRD42020203955.

## Introduction

South and South-East Asian countries comprise one-quarter of the world's population and approximately 150–200 million people have a diagnosed psychiatric disorder and limited access to mental health (1, 2). This has significant health and socio-economic implications; the estimated global loss by 2030 is 12 billion working days and $16 trillion to the economy (3). Despite this huge burden, <1% of national budgets in the South and South-East Asian regions are allocated to mental health (4).

International effort has focussed on reducing the mental health treatment gap in low-middle income countries (LMIC) (5). Acute shortages of qualified mental healthcare workers all levels of the health system significantly contribute to the treatment gap, which is compounded by the centralisation of mental health specialists in urban secondary and tertiary care hospitals (6–10). Although the World Health Organisation (WHO) strongly recommends integrating mental health services into primary care delivery (11), only 30% of South and South-East Asian countries have trained primary care doctors in mental health and only 50% have trained nurses (12).

To address this many LMICs have adopted a task-distribution approach whereby community health workers, with fewer qualifications and a small amount of training, deliver mental health services. However, it still remains necessary to increase the number of highly qualified mental health specialists, namely psychiatrists, psychiatric nurses, psychologists, and clinical psychologists, to manage sicker patients and deliver training, support and supervision to non-specialists.

Education and training of the healthcare workforce is key to reducing the mental health treatment gap and achieving universal health coverage. Our review aimed to describe the various types of educational and training interventions targeted at healthcare workers in South and South-East Asia to improve detection and management of people mental health conditions, their cost- effectiveness, and the enabling factors and barriers that influence the effectiveness of these interventions.

## Methods

### Protocol and Registration

This review was registered in PROSPERO (registration number CRD42020203955) and followed the Preferred Reporting Items for Systematic Reviews and Meta-Analyses (PRISMA-SR) guidelines (13). Ethical approval was not sought for this study as it was an evidence synthesis of existing published research. A detailed research protocol has been previously published (14). Further, there was no deviation from the protocol.

### Review Questions

- What type of educational and training interventions have been used to improve the knowledge, skills, and attitudes of healthcare workers in South and Southeast Asian countries in the early detection and management of people with mental health conditions?
- What type of interventions are effective and for whom are they effective?
- What factors, enabling and barriers, influence how effective these interventions are?
- What type of educational and training interventions are cost-effective?

### Eligibility Criteria

We included studies that focussed on healthcare workers including doctors, nurses, primary healthcare workers, community workers, and lay health counsellors, who are part of the formal health system (Table 1). While there are various permutations and combinations of what constitutes a “formal health workforce,” we have used a widely accepted definition to be individuals employed by the health system to provide direct patient care, in this case mental healthcare, and excluded studies involving volunteers and medical, nursing, or therapy students. We acknowledge that volunteers and students, whilst not a formal part of the healthcare sector, can play a key role (15).

**Table 1:** Eligibility criteria.

**Category**	**Inclusion**	**Exclusion**
Population	Healthcare workers	Volunteers and medical, nursing, or allied health professional students
Intervention	Education, training, and/or capacity building interventions	Interventions that are not education, training, or capacity building
Comparison	Control group with any intervention or usual training or no interventions	None
Outcomes	• Primary: increase detection and improved management of people with mental health conditions • Secondary: improved level of knowledge, skills and attitude	No outcome measures for knowledge of healthcare workers or for mental health conditions

We included studies conducted in countries that are part of South Asia and South-East Asia regions. We used the World Bank's regional definition for South Asian countries (Afghanistan, Bangladesh, Bhutan, India, Maldives, Nepal, Pakistan, and Sri Lanka). We used World Health Organisation's definition for South-East region as they overlap (Afghanistan, Bangladesh, Bhutan, Democratic People's Republic of Korea India, Indonesia, Maldives, Myanmar, Nepal, Pakistan, Sri Lanka, Thailand, and Timor-Leste). We excluded other countries as we sought to reflect cultural and religious similarities in populations.

Interventions had to involve an activity associated with the education, training, or capacity building of healthcare workers to detect and manage mental health conditions. We included all mental health disorders, as defined by the World Health Organization's International Classification of Diseases (ICD)10 (16) and ICD-11 (17) or American Psychiatric Association's Diagnostic and Statistical Manual of Mental Disorders (DSM) IV (18) and DSM-V (19). We included all primary research studies published in English. Abstracts, posters, book chapters, editorials, letters, and secondary research were excluded. Studies published before 2000 were excluded to ensure findings were relevant to current mental health systems.

### Search Strategy

#### Information Sources

We searched six electronic databases on 3 August 2020: Medline, Embase, PsycINFO, Cumulative Index to Nursing and Allied Health Literature (CINAHL), Cochrane, and Global health.

#### Search

The search strategy (Supplementary Table 1) was created using the PICO (population; intervention; comparison; outcomes) framework adapted for each electronic database. We included all primary research studies regardless of the presence of a comparison group to optimise the number of articles identified. To further identify relevant studies, we searched the reference list of all studies selected for inclusion, using forward and backward citation techniques, along with manual searching of journals. We repeated the literature search before data analysis to ensure no recent publications were missed.

#### Study Selection

Using Covidence systematic review software, two independent reviewers (KNK and CB) screened non-duplicate titles and abstracts and conducted full text review. All conflicts at abstract and full-text stage (32% of articles) were resolved by a third reviewer (SG).

#### Data Collection Process and Data Items

Two reviewers (CB, MIK) extracted data independently in Excel and included: country; study design; study setting; study period; sample size; age range; gender distribution; socio-demographic and cultural background; intervention characteristics checklist (Template for Intervention Description and Replication) (20); details of control conditions; outcomes; theory of change; factors influencing implementation of intervention.

#### Risk of Bias in Individual Studies

The risk of bias of each study was critically assessed by two independent reviewers (BC, MK) and disagreements were resolved by review team discussion. We used the Cochrane Collaboration Tool (21) to assess RCT studies and report an overall assessment of bias as “low,” “high,” or “unclear.” The ROBINS-I (Risk of Bias in Non-randomised Studies-of Interventions) tool (22) was used to assess the risk of bias for non-randomised trials and categorise each of the seven domains as “low,” “moderate,” “serious,” “critical,” or “no information.” MMAT (Mixed Method Appraisal Tool) version-8 (23) was used to appraise the quality of qualitative and mixed methods studies; each of the five domains were categorised as “yes,” “no,” or “can't tell.”

## Results

### Study Selection

**Figure 1:**
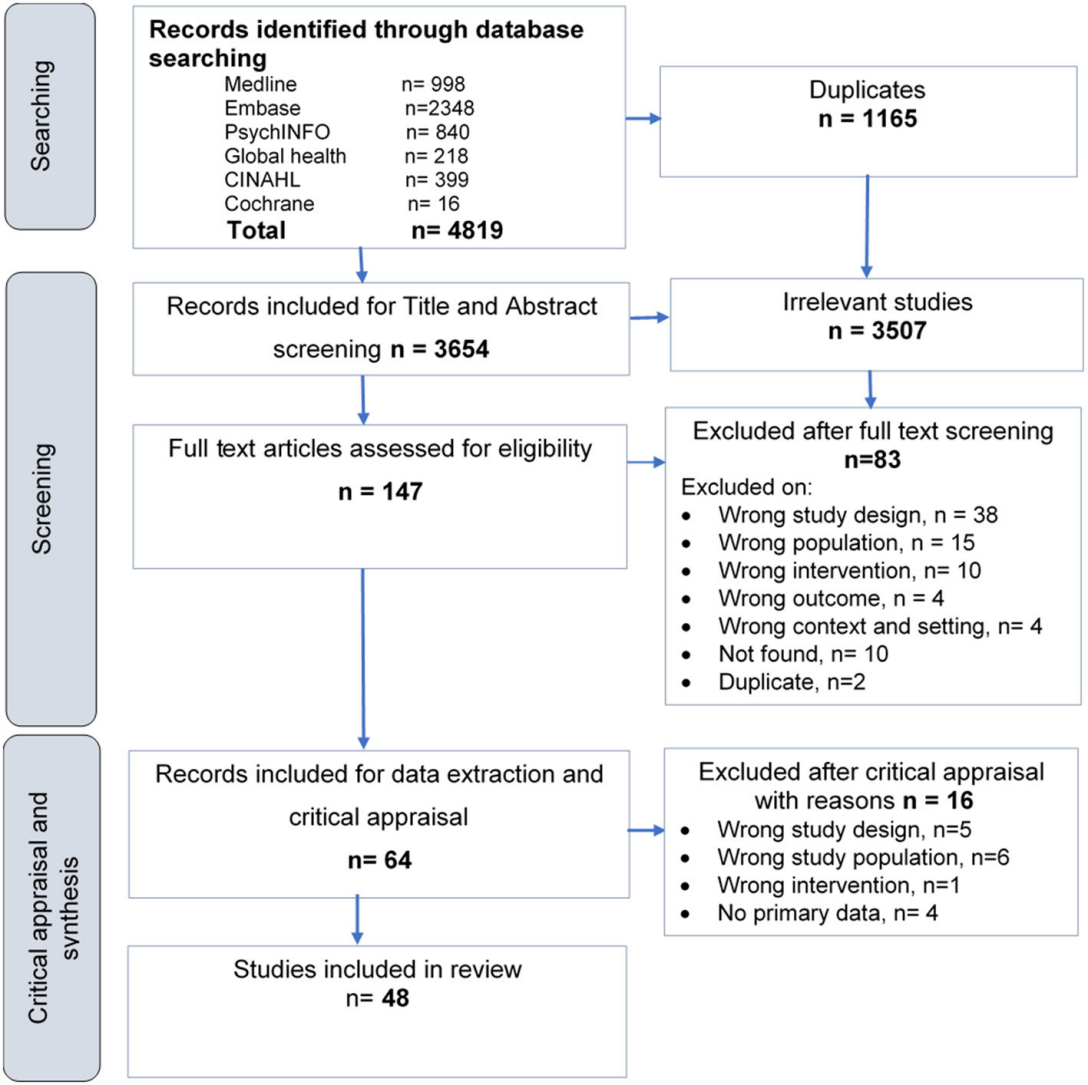
PRISMA flow diagram of included studies.

We screened 3,654 abstracts and 147 full text studies and identified 48 studies that met our eligibility criteria (PRISMA flow diagram, Figure 1).

### Study Characteristics

Of the 48 studies conducted in South and South-East Asia, most studies were undertaken in India (*n* = 17) followed by Nepal (*n* = 10), Pakistan (*n* = 5), Sri Lanka (*n* = 3), Indonesia (*n* = 2), Afghanistan (*n* = 1), and Bangladesh (*n* = 1) (Table 2). No studies were conducted in Bhutan, Democratic People's Republic of Korea, Maldives, Myanmar, Thailand, or Timor-Leste. Nine studies described an intervention with no primary data (Table 3) and were undertaken in India (*n* = 6), Afghanistan (*n* = 1), Bangladesh (*n* = 1), and Sri Lanka (*n* = 1).

**Table 2:** Summary of review studies *(N* = 39).

	**Author, year**	**Setting**	**Study design; total sample size (intervention, control)**	**Description of study**	**Summary of results**	**Quality of evidence/Risk of bias**
**Afghanistan (*****n*** **=** **1)**						
1	Tirmizi et al. (24)	Baharak, Ishkashem, Shughnan & Nusai. Wakhan, Zebak & Maimai, primary care facility	Quasi-experimental; 94 (62, 32)	**Participants:** General medical practitioners, support staff, community health workers **Intervention: WHO mhGAP Intervention Guide & Ministry of Public Health curriculum training** Content: Knowledge on depression, anxiety, PTSD & Drug abuse Duration: 1 month Mode of delivery: Smartphones (using Moodle and Skype) & face-to-face Delivered by: Consultant doctors (Aga Khan Health Service) & researchers (Tech4life Enterprises). **Control:** No information provided	Changes in participant knowledge scores in intervention vs. control sites was 45% pre-intervention to 63% post-intervention test (*p* ≤ 0.001, DF = 61). Average gain in knowledge scores among cases was 16.1 compared to 6.8 in controls.	Moderate
**Bangladesh (*****n*** **=** **1)**						
2	Tarannum et al. (25)	Kutupalong & Nayapara camps	Pre-post-test; 62 (not available)	**Participants:** Psychologists, medical assistants, nurses, medical coordinators, health educators. **Intervention**: **WHO mhGAP-HIG (Humanitarian Intervention Guide)** Content: Mental health conditions, communication skills, human rights of people with mental health conditions, assessment & management of priority conditions Duration: 3 days Mode of delivery: Face-to-face singing, role plays, case studies & video presentations Delivered by: expatriate psychiatrist, Bangladeshi psychiatrist & psychologist **Control:** None	At 7 months, average score of perceived knowledge and skills in health workers pre-post training: 55 vs. 75%. Increased number of consultations from <40 to 160 per month.	Serious
**India (*****n*** **=** **17)**						
3	Maulik et al. (28)	Andha Pradesh, Primary care facility	Quasi-experimental; 45	**Participants:** Primary Care doctors, ASHA workers **Intervention: SMART Mental Health project** Content: Newly developed framework for ASHAs on screening and referral of CMD cases using PHQ-9 and GAD-7 tool and stigma reduction. Doctors were trained using WHO mhGap –IG modules of depression, suicidal intent or self-harm, and other emotional or medically unexplained complaints. Duration: 2-week for ASHAs and 2-h for doctors Mode of delivery: Face-to-face using videos, presentations, and discussions of case vignettes Delivered by: Researchers for ASHA workers One psychiatrist for doctors **Control:** No information provided	• **Increased service uptake by patients: Baseline vs. End line:** 3.3 vs. 81.2% (odds ratio 133.3, 95% CI 89.0 to 199.7; *P* < 0.001). • **Symptom reduction: Baseline vs. End line:** Depression = 13.4 to 3.1% (*P* < 0.001) and Anxiety = 12.9 to 1.9% (*P* < 0.001),	Moderate
4	Ashok et al. [26]	Kashmir, primary care facility	Pre-post-test; 40 (not available)	**Participants**: Lay health workers **Intervention: Pre-post mental health training program** Content: Basic mental health services for severe mental illnesses in a conflict area Duration: Unavailable Mode of delivery: Unavailable Delivered by: Psychiatrist, psychologist & social worker **Control:** None	At 14 months trained lay health workers: • 279 patients were identified during the 14 months of the project than those in the preceding 2 years and during consultation, 262 of these were diagnosed by a psychiatrist. At 12 months, 205 patients (78%) remained engaged with the service and perceived it as very helpful. • Repeated measures ANOVA showed significant improvements in scores: patients' symptoms and personal functioning (GAF): *F* [df 3.449] = 104.729, *p* =0.001)	Moderate
5	James et al. (27)	Karnataka, primary care facility	Pre-post-test; 95 (not available)	**Participants:** ASHA workers **Intervention: Pre-post mental health training** Content: Diagnosis & treatment of severe mental illness using ‘symptoms in others' tool & referral of severe cases. Activists) workers Duration: 1 day Mode of delivery: Face-to-face Delivered by: Psychiatrist **Control:** None	Changes at detection of mental illness related patients by the health workers: • Detection person with mental illness by ASHA workers (CAMI): 65/95 community healthcare workers personally brought a person with mental illness for treatment to one of the PHC.	Moderate
6	Muke et al. (29)	Sehore, Madhya Pradesh, primary care facility	Qualitative: focus group discussion; 32 (not available)	**Participants:** ASHA workers **Intervention: Training on depression** Content: Psychological knowledge development, screening for depression, introduction to counseling and how it differs from a friendly chat Duration: 1 day Mode of delivery: Mobile, laptop & tablets Delivered by: Research team **Control:** None	Participants identified the following key areas: • Training on detection and treatment of depression was considered important to address “stress” and “tension” within their communities. • Despite limited familiarity with using digital technology, the platform was viewed as useful and convenient. • Simple language for the program and uses of interactive content and images to increase interest and improve engagement.	Low
7	Mehrotra et al. (30)	Chattisgarh,primary care facility	Pre-post-test; 12 (not available)	**Participants:** Counselors, clinical psychologists, psychiatric social workers **Intervention**: **Online & app-based learning** Content: Screening of major mental illness, working with families, discussion on relapse prevention, motivational interviewing & grief therapy Duration: 10 days (2 days per month)Mode of delivery: Online Delivered by: National Institute of Mental Health and Neuro Sciences Bengaluru ECHO **Control:** None	Changes in knowledge, self-efficacy and self-confidence of health workers: • Improvement of knowledge on CMD: Changes between pre-test and post-test: *t*-test: 3.71, *p* ≤ 0–01 • Improvement in perceived self-confidence and self-efficacy: mean score of Pre-test: 58.16, (SD = 12.23) vs. post-test: 70.83, (SD = 11.06) • Improvement in treatment performance (HCW): 12 counselors had provided clinical services to 4743 patients in 6 months.	Moderate
8	Michael et al. (31)	Rural Bangaluru, primary care facility	Qualitative; 5 (not available)	**Participants:** Community health workers **Intervention: Training on geriatric mental health** & qualitative interview for need assessment for medical interventions Content: Prevention, promotion, risk factors & identification of: depression, dementia, anxiety disorders, substance abuse & interventions Duration: Unavailable Mode of delivery: Face-to-face Delivered by: Unavailable **Control:** None	Health workers gained knowledge of common mental health issues in old age. The need for medical interventions was recognized and knowledge about psychosocial interventions.	Critical
9	Maulik et. al. (32)	Andhra Pradesh, primary care facility	Pre-post-test; 23 (not available)	**Participants:** General medical practitioners & community health workers **Intervention: SMART Mental Health project and use of WHO mhGAP guideline** Content: Screening & treatment (drugs & counseling) of CMDDuration: 10 days Mode of delivery: Face-to-face Delivered by: Principal investigator, research & field staff **Control:** None	Effect of health workers intervention on patients: There was a significant reduction among the patients in mean score of depression by 3.6 (SE 0.6, *P* < 0.0001) and a reduction in anxiety mean score by 1.3 (SE 0.4, *P* = 0.004) at the end of intervention.	Serious
10	Shidhaye et al. (33)	Rural Sehore-Madhya Pradesh,primary care facility	Mixed method; 35 (not available)	**Participants:** Primary & community health workers, health service providers, paramedical, social workers, state & district-level planners **Intervention: Programme for Improving Mental health care (PRIME) training program** Content: District integrated mental healthcare plan to detect & manage depression, psychosis & alcohol use disorders Duration: 2 days Mode of delivery: Face-to-face Delivered by: Multi-disciplinary experts **Control:** None	A very small number of patients being detected and started with treatment for priority mental disorders: • At 1 month of follow-up, 7 patients were detected and 3 patients were referred to the district hospital by 7 medical officers (depression *n* = 5; psychosis *n* = 1; alcohol *n* = 1) • Basic psychoeducation was provided to 4 patients with depression (the remaining being directly referred to the district hospital). • No pharmacological treatment was provided to any of these patients by the medical officers as none of the psychotropic medications on the essential drug list were available in the facility. The front-line workers (accredited social health activists, volunteers with similar roles as community health workers) were also followed-up on a weekly basis in the first month following the training; none of them had identified or referred any patient to the facility.	High
11	Nimgaonkar et al. (34)	Tamil Nadu, primary care facility	Pre-post-test; 106 (not available)	**Participants:** Village health workers, general medical practitioners, nurses, health animators **Intervention: Pre-post mental health training program** Content: Knowledge on basic mental illness symptoms Duration: Not available Mode of delivery: Face-to-face lectures, video clips of patients before & after treatment, role-play, monthly team reviews & supplementary workshops Delivered by: Academic psychiatrists **Control:** None	Follow-up at end of 3-year mental health program. Changes among the health workers and patients after given the intervention: Pre-posttest knowledge and attitude of healthcare workers: 35 vs. 94% Daily functioning of patient (based on 3-point scale): Mean ± standard deviation; initial visit: 2.38 ± 0.77, last visit: 2.56 ± 0.70, *N* = 157; *p* = 0.01, paired *T*-test Proportion of self-referrals increased from 27 to 57% over 3 years. Salaries for healthcare workers account for 36.5% of costs, while medications accounted for 27.4%. Inpatient care accounted for only 2.1% of the total, consistent with the emphasis on community care. Approximately 23.3% of the budget was allocated to capacity building and training. The annual per capita expenditure was 1635,146.66 Rupees (27,132.60 US dollars).	Moderate
12	Paudel et al. (35)	Rural Jamkhed, primary care facility	Qualitative: focus group discussion; 26 (not available)	**Participants:** Village health workers **Intervention: Training on depression & anxiety** Content: Symptoms & management of depression & anxiety Duration: Unavailable Mode of delivery: Face-to-face Delivered by: Social workers **Control:** None	• 4 groups were able to diagnose the presented case of depression correctly. • Community health workers were able to identify many symptoms and to suggest management options for depression, • They were supportive and empathetic toward people with depression and could coordinate care with other existing health team and community people.	Low
13	Beck et al. (36)	Chennai Primary care facility	Pre-post-test; 14 (not available)	**Participants:** Non-mental health professionals **Intervention: Pre-post training on depression** Content: CBT & use of peer supervision Duration: 5 days Mode of delivery: Face-to-face Delivered by: Clinical psychologist. **Control:** None	Indian and UK health workers scores were compared Knowledge on understanding CBT (MPQ-PC): UK (9) vs. Indian average (9.2) Knowledge on clinical application of CBT (PCQ-PC): UK (16.3) vs. Indian average (18.7)	Critical
14	Gupta et al. [39]	No further information	Pre-post-test; 20 (not available)	**Participants:** Psychologists, general medical practitioners, consultants (not specified)**Intervention: Training on CBT (Cognitive Behavioral Therapy)** Content: CBT model & rationale, patient suitability, supervision & behavioral experiments. Duration: 3 days Mode of delivery: Face-to-face role play & supervision over telephone, video chat & email Delivered by: Clinical psychiatric specialist **Control:** None	Improvement in knowledge among health workers on CBT: pre and post-test: *t*-test; *t*_(13)_ = −6.899, (*p* ≤ 0.001, *r* = 0.886)	Serious
15	Armstrong, et al. [40]	Karnataka, primary care facility	Pre-post-test; 66 (not available)	**Participants:** Junior health assistants, village rehabilitation workers, ASHA workers of NGO Gramina Abrudaya Seva Samstha (GASS) **Intervention: Pre-post mental health training program** Content: Identification of mental disorders, mental health first aid, practice-based skills, enhancing appropriate response and referral, support people with mental disorders and their families, and improving mental health promotion in communities Duration: 4 days Mode of delivery: Face-to-face Delivered by: Local health professionals **Control:** None	Changes among health workers: • Improvements in identification of depression: Pre vs. post-test: 22.7%, (95% CI = 13.3–24.7, *p* = 0.002) vs. 50.0%, (95% CI = 37.4-62.7, *p* = 0.002) and at 3 months follow-up: 43.9%, (95% CI = 31.7–56.7) • Improvements in identification of psychosis: Pre vs. post-test: 9.1%, (95% CI = 3.4–18.7, *p* = 0.001) vs. 27.3%, (95% CI = 10–39.6, *p* = 0.001) and at 3 months 34.8%, (95% CI = 23.5–47.6) • Reduction of stigmatizing attitudes to people with mental disorders: Decreased from 84.8 to 62.1%	Serious
16	Jincy et al. (37)	Mangalore & Karnataka, tertiary care facility	Pre-post-test; 35 (not available)	**Participants:** Nurses **Intervention: Pre-post training program on care & suicide prevention** Content: Suicide prevalence, myths, misconceptions, risk factors, prevention & nursing care of attempted suicide patients Duration: Unavailable. Mode of delivery: Face-to-face using a booklet Delivered by: psychiatrist, associate professor, lecturer, postgraduate student & mental health nursing department **Control:** None	Changes among the nurses: • Improvement of knowledge on suicide: Changes between pre and post-test: *Z* = 5.183, *p* ≤ 0.001 • Improvement of attitude toward attempted suicide patients: changes between pre and post-test: *Z* = −4.380, (*p* ≤ 0.001) • Positive correlation between pre-test knowledge score and pre-test attitude score (correlation coefficient was 0.434, significant at 0.01 level)	Critical
17	Patel et al. (38)	Goa, primary care facility	RCT; 2796 Public: (823, 825) Private: (537, 611)	**Participants:** Lay health counselors **Intervention: MANAshanti Sudhar Shodh (MANAS) intervention** Content: Psychoeducation, common mental disorders, interpersonal difficulties, sharing emotional symptoms with doctor & caring family members or other key persons in their social networks, strategies for symptom alleviation (e.g., breathing exercises for). Treatment adherence, providing information about social/welfare agencies Duration: 2 months Mode of delivery: Face-to-face Delivered by: Psychiatrist **Control:** Physicians in usual care	Beneficial effects at public facilities at 12 months but not among participants attending private facilities. • Suicide attempts/plans: risk ratio = 0.64 (95% CI: 0.42 to 0.98) • Psychological morbidity (CIS-R): −3.89 (95% CI: 77.8 to 0.03), *p* = 0.05 • Prevalence of common mental disorders: risk ratio = 0.70 (95% CI: 0.53 to 0.92)	Low
18	Thara et al. (39)	Rural Cuddalore & Pondicherry, primary care facility	Qualitative; 127 (not available)	**Participants:** Community level workers **Intervention: Psychosocial training program** Content: Management of psychological problems focusing entirely on disaster management and merely glossing over psychological issues. Duration: 2–3 days Mode of delivery: Face-to-face through discussion, audiovisual and role-play Delivered by: Mental health specialist **Control:** None	Participants were asked to recall their experiences of psychosocial training 2 years previously.	Low
19	Shaji et al. (40)	Kerala, tertiary care facility	Pre-post-test; 19 (not available)	**Participants:** Angawadi community health workers **Intervention: Pre-post training on Dementia Content:** Dementia; its clinical features, causes, and prognosis Duration: Not available Mode of delivery: Face-to-face through case vignettes and discussion Delivered by: Local academic psychiatrist. **Control:** None	Changes in detection of dementia patients by trained health workers: • Community health workers identified 5/ 1979 suspected dementia cases. • Following the psychiatrist's assessment, 33 met DSM-IV criteria for dementia, making the positive predictive value of the community health workers informal screening 64.7%.	Moderate
**Indonesia (*****n*** **=** **2)**						
20	Anjara et al. [43]	Yogyakarta, Primary Care Facility (Pukesmas)	RCT; 28 (14, 14)	**Participants:** General medical practitioners, nurses **Intervention: WHO mhGAP Intervention Guide** Content: Detection and management depression, anxiety, panic attack, obsessive compulsive disorder, social & agora phobia Duration: 1 month Mode of delivery: Face-to-face Delivered by: clinical psychologists & psychiatrists **Control Participants:** Clinical psychologists **Intervention :** Content: Basic and advanced psychosocial therapies to manage disorders. Duration: Not available Mode of delivery: Face-to-face Delivered by: Not available	After 6 months of the intervention given to patients. No significant difference between trained GPs and nurse's vs. psychologists in managing mental health conditions. Used CISR diagnostic tool to examine the remission rate: Chi-square = 0.164, df = 1, *p* = 0.730 at 6 months. Clinical psychologist vs. general medical practitioners and nurses were more cost-effective. Cost-effectiveness analysis using QALYs indicate the 50% probability of care by a clinical psychologist co-located in primary care being more cost-effective than GPs at the Indonesian willingness to pay for medical interventions	High
21	Theresia et al. (41)	Indonesia, Primary care facility	Mixed method; 16 (not available)	**Participants:** Non-specialist resident doctors, psychiatrists, psychologists, social workers **Intervention: Training on stress management among children and adolescents of disaster-prone areas** Content: Potential effects, resilience development, identification of community resources, comprehensive management; including Psychological First Aid, crisis intervention and stress management among children and adolescent Duration: 5 days Mode of delivery: Face-to-face throughdiscussions, case studies & roleplays Delivered by: Multidisciplinary team of 8 staff from psychiatric division **Control:** None	Improvements in knowledge Pretest: mean= 13/20 (SD = 2.06) vs. post-test mean= 15.8 (SD = 1.6) (79%, SD = 8.19%). Differences between pre-test and post-tests improvements in knowledge (mean = 2.75, 13.75%) were statistically significant (*p* < 0.001). Focus group discussion found benefits of active learning and use of case studies and roleplays. Early detection especially using the Strengths and Difficulties Questionnaire, case formulation, and comprehensive management, including Psychological First Aid (and crisis intervention were helpful).	Low
**Nepal (*****n*** **=** **10)**
22	Aldridge et al. (42)	Chitwan, primary care facility	Case-control; **Depression:** 212 (137,75) **AUD:** 232 (175,57)	**Participants:** Healthcare workers **Intervention: The Programme for Improving Mental Health Care (PRIME); WHO mhGAP Intervention Guide training** Content : Psychotropic medication, psychosocial support, or psychoeducation Duration: Unavailable Mode of delivery: Face-to-face through mhGAP Intervention Guide Delivered by: Non-specialist prion **Control:** Participants received standard primary care at the health facilities consisting of assessment, diagnosis, and treatment of somatic conditions, with no mental health treatment provided.	Suicidal ideation at baseline and 12 months relative to comparison cohorts • Depression cohorts: OR = 0.31, 95% CI: 0.08–1.12; *p* = 0.07), with a significant effect of treatment over time (*p* = 0.02). • Alcohol use cohorts: OR = 0.46, 95% CI: 0.06–3.27; *p* = 0.44), and there was no effect of treatment over time (*p* = 0.72).	Moderate
23	Gupta et al. (43)	East, primary care facility	Pre-posttest; 49 (not available)	**Participants:** Community health workers **Intervention: Training on diagnosis and management of depression and anxiety** Content: Diagnosis and management of depression, anxiety and psychoeducation Duration: Not available Mode of delivery: Face-to-face through presentation, role plays & question-answer session Delivered by: Author of workshop **Control:** None	Self-reported changes among community health workers at 1- & 3-months post-training (reported results do not distinguish between time points): • 69% improvement in knowledge on psychoeducation and counseling • 84% improvement in skills and confidence on psychoeducation and counseling. • 27% reduction in need to refer patients with depression and anxiety • Acceptability: the overall impression of the training was satisfactory. Participants stated need for regulartraining to update knowledge and skills	Serious
24	Jordans et al. (44)	Chitwan, primary care facility	RCT; 196 (105, 91)	**Participants:** Community health workers **Intervention: The Programme for Improving Mental Health Care (PRIME); Community Informant Detection Tool (CIDT)** Content: Detection and referral of depression, psychosis & alcohol use disorder, basic training on mental health, understanding of the referral system & stigma. Duration: Unavailable Mode of delivery: Unavailable Delivered by: Unavailable **Control:** 2 days face-to-face basic training on mental health, understanding of the referral system & stigma.	Median number of patients identified with mental illness using routine health facility data was 47% greater (*p* = 0.04, *r* = 0.42) in intervention (*n* = 24/309) vs. control arm (*n* = 16/182) at 6 months post training.	Low
25	Kohrt et al. (45)	Chitwan, tertiary care facility	Pre-posttest; 41 (not available)	**Participants:** Primary health workers, health assistants, community medical assistants, auxiliary nurse midwives **Intervention: The Programme for Improving Mental Health Care (PRIME); Reducing Stigma among Healthcare Providers (RESHAPE)** Content: Theoretically-grounded intervention to tackle primary healthcare providers' stigma against persons with mental illness. Duration: Unavailable Mode of delivery: Face-to-face Delivered by: Unavailable **Control:** None	Changes among the healthcare providers at 16 months [median (interquartile range, IQR)]: • Willingness to interact with a person with mental illness (SDS): increased from 54 to 81% [Prescribers: 32.5(19.2, 47.0) to 15.0(12.0, 21.0), z = −3.1, *p* = 0.002; Non-prescribers: 28.8(21.6, 40.8) to 17.0(13.0, 29.0), z = −2.3, *p* = 0.02]. • Clinical competence (ENACT): increased from 49% pre-training to 93% [Prescribers: 30.0 (26.0, 32.0) to 47.0(35.0, 50.0), z = 3.5, *p* < 0.001; Non-prescribers: 26.0(26.0, 27.0) to 45.0(38.0, 49.0), z = 3.4, *p* < 0.001] • mhGAP Knowledge: [Prescribers: 60.0 (60.0, 70.0) to 83.3(73.3, 86.7), z = 3.6, *p* < 0.001; Non-prescribers: 64.0(52.0, 72.0) to 74.0(68.0, 76.0), z = 2.7, *p* = 0.007] • mhGAP Attitudes: [Prescribers: 1.7(1.6, 1.8) to 1.4(1.3, 1.5), z = −3.3, *p* = 0.001; Non-prescribers: 32.0(27.6, 35.0) to 37.0(33.0, 39.0), z = −3.4, *p* < 0.001]	Low
26	Luitel et al. (46)	Rural Chitwan, primary care facility	Mixed methods; 35 (not available)	**Participants:** Health workers **Intervention: The Programme for Improving Mental Health Care (PRIME); Implementation of mhGAP guide** Content: I) Health organization level: Mental health program coordination, policy for provision, referral system in district hospital, ii) Health facility level: Awareness and anti-stigma training, protocol and guidelines, psychotropic treatment iii) Community level: Mass community sensitization, improve treatment coverage, community dictation, advanced psychosocial counseling, home-based care Duration: Unavailable Mode of delivery: Unavailable Delivered by: Unavailable **Control:** None	Changes among the health workers: • The proportion of primary care patients that received mental health services increased by 1200% over the 3-year implementation period. • Barriers: frequent transfer of trained health workers, lack of confidential space for consultation, and no mental health supervision in the existing system, and stigma. • Facilitators: Involvement of Ministry of Health, procurement of new psychotropic medicines through PRIME, motivation of health workers and the development of a new supervision system. • Paired *t*-tests: Knowledge (mhGAP adapted): 3.89, *p* < 0.001 Stigma (MICA& mhGAP adapted): 3.05, *p* 0.004; −3.86, *p* < 0.001 clinical competencies (ENACT): 7.8, *p* < 0.001 *mhGAP self-efficacy adapted for PRIME: 16.46, p <0.001*	High
27	Sangraula et al. (47)	Sindhuli, primary care facility	Pilot RCT; 130 (61, 60)	**Participants:** Community-based psychosocial health workers **Intervention: Problem management Plus in mental health** Content: Depression, psychosocial distress & PTSD, how to manage stress and change behavior. Duration: 10 days Mode of delivery: Unavailable Delivered by: Unavailable **Control**: Enhanced usual care consisting of family meetings discussing psycho-social support & provision of information on mental health services.	7 days after a patient received support. Reduction in: • Depression (PHQ-9) in intervention (−3.5, SD = 4.3) vs. control (−1.6, SD = 3.4) group. • Common mental disorders (GHQ-12) in intervention (−12.3, SD = 7.5) vs. control (−3.7, SD = 7.0) group. • Common psychosocial problems (PMPH) in intervention (−1.0, SD = 2.8) vs. control (−0.1, SD = 2.7) group • Post-Traumatic Stress Disorder (PCL-5) in intervention (−2.7, SD = 7.0) vs. control group (−1.3, SD=5.6).	Low
28	Markkula et al. (48)	Dang, primary care facility	RCT; 287 (141, 146)	**Participants:** Non-medical healthcare workers **Intervention: Psychosocial counseling** Content: Psycho-social counseling, basic therapeutic skills, components of CBT, problem-solving, exposure therapy, yoga & meditation. Duration: 5 days Mode of delivery: Face-to-face Delivered by: Unavailable **Control:** Enhanced usual care Primary health workers received 5 days training and 3 days refresher training on detection and treatment of mental health disorders.	Patient care with depression & anxiety by non-medical healthcare workers at 6 months. Significant differences: • Depression: BDI mean score difference between intervention and control arm:−7.43 (95% CI: −9.71 to −5.14, no *p*-value) • Anxiety: BAI mean score difference between intervention and control arm: −5.42 (95% CI: −7.59 to −3.27, no *p*-value).	Low
29	Kohrt et al. (49)	Nepal (Uganda & Liberia)tertiary care facility	Pre-post-test; 44 (not available)	**Participants:** Primary care workers, community health workers, social workers **Intervention: The Programme for Improving Mental Health Care (PRIME); WHO mhGAP-Intervention Guide in humanitarian conflict settings** Content: Identification and treatment of epilepsy, psychosis/mania, and depression, reduction of stigma and discrimination; pro-active case finding; homebased care; and patient support groups, communication skills, collaborative care, community-based components and case detection Duration: Unavailable Mode of delivery: Face-to-face Delivered by: Psychiatrists trained in mhGAP & Psychosocial counselors **Control:** None	Changes in competency post-training (paired *t*-test): • Competency related to depression and epilepsy (ENACT): 26% (SD = 13%, *n* = 30) to 60% (SD = 18%, *n* = 31; *t* = 8.38, *p* < 0.001). Changes in attitudes (stigma) and knowledge pre-refresher course: • Stigma: (MICA & SDS): −3.25 (*p* = 0.003) d −0.55 & 0.60 (p 0.003) d −0.55 • mhGAP knowledge: 10.37 (<0.001) d 1.75 mhGAP attitudes:−2.46 (*p* = 0.02) d −0.42	Serious
30	Acharya et al. (50)	Accham, primary care facility	Pre-posttest; 27 (not available)	**Participants:** General medical practitioners, psychosocial counselors, community medical assistants, health assistants **Intervention: Pre-post mental health training** Content: Skills-building & clinical coaching on depression, psychosis & PTSD. Duration: 5 day + 7 days Mode of delivery: Face-to-face video lectures Delivered by: 1 Psychiatrist **Control:** None	Changes in health workers knowledge pre-test vs. post-test (median scores) • Depression: 89 vs.11, (*p* = 0.12) • PTSD: 60 vs. 20, (*p* = 0.01) • Acute stress reaction: 40 vs. 20, (*p* = 0.03) • Grief: 60 vs. 40, (*p* ≤ 0.01) • Psychosis: 67 vs. 22, (*p* = 0.01)	Critical
31	Jordans et. al. (51)	Chitwan, Pyuthan, tertiary care facility	Pre-posttest; 509	**Participants:** Female community health workers (called volunteers)**Intervention: Community Informant Detection Tool (CIDT)** Content: Detection & referral of mental disorders to healthcare facilities, ethics, confidentiality, encourage help-seeking behavior, & pharmacological & psychosocial treatment. Duration: 2 days Mode of delivery: Face-to-face Delivered by: NGO **Control:** None	Number of individuals referred to a healthcare facility using the CIDT tool: • 509 individuals identified • 341 (67%) accessed a health-care facility after being referred • 264 (77%) started mental health treatment • People in the rural Pyuthan district (208 out of 268) were more likely to access health care than those living in Chitwan district (133 out of 241)	Serious
**Pakistan (*****n*** **=** **5)**						
32	Rahman et al. (52)	Swat, primary care facility	RCT; 80 (40, 40)	**Participants:** Female health supervisors **Intervention: Thinking Healthy programme – master training model** Content: CBT for perinatal depression using Technology-Assisted Cascaded Training and Supervision system Duration: Unavailable Mode of delivery: Online Delivered by: CBT psychologist **Control:** CBT psychologists deliver 5 days training to community health workers.	At 3 months no significant differences in mean competency scores between intervention vs. control arm • Competence of community health workers (ENACT) after the intervention:24.97 vs. 27.27 (95% CI: 4.87 to 0.27, *p* = 0.079) • Mean score of competencies (ENACT) after 3 months = 44.48, SD = 3.97 v 43.63, SD = 6.34, p = 0.53 (95% CI: 1.88 to 3.59). Training and supervision costs per community health worker was $54 less in digital arm ($117)	Low
33	Humayun et al. (53)	Bannu district, North Waziristan, Primary care facility	Pre-posttest; 56 (not available)	**Participants:** General medical practitioners & psychosocial health workers **Intervention: WHO mhGAP-IG training programme** Content: Diagnosis, treatment, follow-up & referral of neurological and substance use disorders, depression, psychosis, child & adolescent mental health (includes learning disability), epilepsy & drug dependence Duration: 2 days Mode of delivery: Face to face through large and small group discussions, individual exercises Delivered by: 3 psychiatrists **Control:** None	Perceived knowledge of health workers: mean pre- (15.43, 62% (*p*-value 0.000, S.D.4.05) vs. post- (19.48, 78% (*p*-value 0.000, S.D. 3.13) test.	Moderate
34	Zafar et al. (54)	Rural Rawalpindi, primary care facility	Qualitative; 40 (not available)	**Participants:** Lady health workers **Intervention: THP (Thinking Healthy Programmer) with CBT (Cognitive Behavior Therapy) and SPRING (Sustainable Programme Incorporating Nutrition and Games)** Content: Integration of THP with CBT principles for anxiety, depression & marital distress and maternal psychosocial well-being integration in SPRING. Duration: 6 days Mode of delivery: Face-to-face Delivered by: Not available **Control:** None	4 focus group discussions were conducted: Participants could apply skills gained from the training effectively to their work, and the approach was found to be useful by CHWs, mothers, and their families. Success of approach attributed to mothers being the central focus of the intervention, using local CHWs whom mothers trust, simplified training and regular supervision, and an approach that facilitates, not adds, to existing CHWs' work.	High
35	Taj et al. (55)	Rural, primary care facility	Pre-post-test; 50 (not available)	**Participants:** Mental health professionals (not specified)**Intervention: Pre-post mental health training program** Content: Multi-method teaching on practical skills acquisition on depression, anxiety, substance abuse and other common problems Duration: 5 days Mode of delivery: Face-to-face through slides, role-plays and video-showing Delivered by: Unavailable **Control:** None	Changes in knowledge of health workers: Perceived knowledge on depression, anxiety, and substance use disorder: Pre-test total score= 3130 (Average = 65%) vs. Post-test total score = 3624 (Average = 74%)	Serious
36	Rahman et al. (52)	Rawalpindii, primary care facility	RCT; 40 (20, 20)	**Participants:** Community health workers **Intervention: Cognitive behavior therapy (CBT)** Content: Use of CBT techniques to treat perinatal depression Duration: 3 days Mode of delivery: Face-to-face Delivered by: Unavailable **Control:** Enhance routine care (structured and monitored care in the community) and trained female health workers visited pregnant women routinely for 4 weeks.	Adjusted mean difference between intervention vs. control at 12 months: • Depression (HDRS): −5·86 (95% CI: −7·92 to −3·80) and −6·65 (95% CI: −8·56 to −4·74) • Disability (BDQ): −1·80 (95% CI: −2·48 to −1·12) and −2·88 (95% CI: −3·66 to −2·10) • Daily functioning (GAFS): 6·85 (95% CI: 4·73 to 8·96) and 8·27 (95% CI: 6·23 to 10·31) • Perceived social support (MSPSS): 6·71 (95% CI: 3·93 to 9·48) and 7·85 (95% CI: 5·43 to 10·27)	Low
**Sri Lanka (*****n*** **=** **3)**						
37	Siriwardhana et al. (56)	Northern province, primary care facility	Pre-post-test; 12 (not available)	**Participants:** Primary care practitioners **Intervention: WHO mhGAP Intervention Guide** Content: Depression, stress related disorders, medically unexplained symptoms, alcohol/drug disorder & suicide Duration: 3 days Mode of delivery: Face-to-face through video materials Delivered by: local psychiatrist & mhGAP trained trainer **Control:** None	Changes in knowledge on mhGAP post training. Mean pre- and post-test scores on key modules of mhGAP guide: 72.8 % (SD; 11.4) and 77.2 % (SD; 11.8) respectively, and pre/post score difference was statistically not significant (paired *t* test; *t*_(9)_ = −1.408, *p* = 0.193).	Serious
38	Blignault et al. (57)	Primary care facility	Pre-post-test; 90 (not available)	**Participants:** Phase I: Eight Senior clinicians Phase II: Medical officers of mental health (MOMH), psychiatric support workers andgeneral counselors **Intervention: Training program on mood disorders** Content: Conceptual and diagnostic issues, clinical application, community aspects, public policy Duration:Phase I: 14 + 5 days Phase II: 1.5 days Mode of delivery: Face-to-face Delivered by:Phase I: Expert professionals Phase II: Trained senior clinicians **Control:** None	Changes of the health workers on how to manage mood disorders as assessed on a set of short-answer questions: • Knowledge: *n* = 44, 4.5 ± 2.5 vs. 6.2 ± 2.0, CI = 0.86–2.71, *p* = 0.0002. • Attitude (MICA): *n* = 39, 36.9 ± 6.9 vs. 32.4 ± 7.2, CI = 2.82–6.15, *p* = 0.0000 • CBT training: 85% participants thought workshop helped to understand CBT principles, 63% thought they would be able to help their patients with CBT techniques. 33% thought this would help them with their own problems, while 37% indicated that they would consider applying CBT principles in the management of medically unexplained symptoms.	Serious
39	Jenkins et al. (58)	Colombo, Kandy, Jaffna, Batticoloa & Galle, primary care facility	Pre-post-test; 260 (not available)	**Participants:** Psychiatrists, general medical practitioners **Intervention: Pre-post mental health training program** Content: Depression, psychosis and somatization; on core concepts of mental health disorders and its detection and management; core skills (communication skills, assessment, mental state examination, diagnosis, management); Common neurological disorders (epilepsy, Parkinson's disease, headache, dementia, toxic confusional states); Psychiatric disorders (content based on the WHO primary care guidelines for mental health); and System issues of policy (legislation)Duration: 5 days Mode of delivery: Face-to-face through discussion, role-plays and World Psychiatric Association videos Delivered by: Unavailable **Control:** None	Changes among the health workers post training. Perceived knowledge of participants: mean scores pre- and post-training who completed the tests: Colombo: 66.4 vs. 82.0 %, (*n* = 32) Kandy: 74.0 vs. 90.0%, (*n* = 46) Jaffna: 62.8 vs. 67.4%, (*n* = 35) Batticaloa: 69.0 vs. 77.0%, (*n* = 51) Galle: 74.9 vs. 76.4%, (*n* = 20)	Critical

**Table 3:** Summary of articles with description of intervention only (*N* = 9).

**References**	**Country; setting**	**Description of training and capacity building intervention**
Lakshminarayanan et al. (59)	India; Bangalore, Khed, Khagaria, & Sahibganj, primary care facility	• **Participants:** Lay counsellors • **Intervention:** • Content: Perinatal mental health (preventative and promotive approaches, screening, referrals, facilitating mental health literacy, engaging families and communities to deliver mental health services for pregnant mothers) • Duration: 1 month • Mood of delivery: Online video conferencing, instant messaging and screen sharing • Delivered by: Multi-disciplinary team of health professionals. • **Control:** None • No further information is provided
Jayaram et al. (60)	India; Mugalur rural primary care facility	• **Participants:** Community health workers • **Intervention:** • Content: Screening and treatment of common medical conditions, and mental health stigma by street play • Duration: Unavailable • Mood of delivery: Face-to-face • Delivered by: Psychiatrists and medical social workers • **Control:** None • No further information is provided
Malhotra et al. (61)	India; Chandigarh tertiary care facility	• **Participants:** Psychologists, social workers, general medical practitioners and psychiatrists • **Intervention:** • Content: Diagnosis and management of mental health disorders (depression, psychosis, anxiety, substance abuse) using Clinical decision support system (CDSS) tool • Duration: 2 days • Mood of delivery: Face-to-face sessions and online video-conferencing using Skype • Delivered by: Psychologists who were supervised by psychiatrists at Postgraduate Institute of Medical Education and Research • **Control:** None • No further information is provided
Varghese et al. (62)	India; south tertiary care facility	• **Participants:** Nurses • **Intervention:** • Content: Identification and management of delirium • Duration: 42 days • Mood of delivery: Unavailable • Delivered by: Unavailable • **Control:** None • No further information is provided
Chowdhury et al. (63)	India; rural West Bengal primary care facility	• **Participants:** Community health workers • **Intervention:** • Content: Identifying mental health illness (schizophrenia, depression, anxiety and depression, epilepsy, harmful effects of alcohol, and drug dependency and domestic violence) and to provide counselling and monitoring • Duration: 5 days • Mood of delivery: Face-to-face • Delivered by: Multi-disciplinary team of doctor, consultant, and clinical psychologist • **Control:** None • No further information is provided
Chowdhury et al. (64)	India; Sundarban, Majarajganj, Kolkata tertiary care facility	• **Participants:** Local Healthcare Providers, Integrated Child Development Scheme workers, multi-Purpose healthcare workers • **Intervention:** • Content: Mental health and illness including self-harm, symptoms of common neuro-psychiatric disease, counselling, and monitoring suitable cases • Duration: 2 days • Mood of delivery: Face-to-face • Delivered by: a consultant psychiatrist, a trainee psychiatrist and a psychologist • **Control:** None • No further information is provided
Mahmuda et al. (65)	Bangladesh; Cox's Bazar Rohingya refugee camps	• **Participants:** Psychology students with 6-months mental health experience • **Intervention:** • Content: Group Integrative adapt therapy(IAT) on refugee mental health problems, psychological and psychosocial treatments, adaptation and integration of ADAPT model: reducing the gap between individual and psychosocial responses in refugees, IAT background, theory and adaptation among the Rohingya Muslims, knowledge on IAT components and administration, how to test the IAT with Rohingya refugees to assess the feasibility and acceptability of IAT, cultural adaption on mental health and psychosocial assessment tools, safety management for field personnel, key ethical issues professionalism and fidelity of intervention trials with refugees and gender and intimate partner violence among the refugees • **Intervention:** Duration: 10-day • Mood of delivery: Face-to-face classroom based with field visits • Delivered by: Developer of Group Integrative Adapt Therapy (IAT), advisor of UNHCR, and wo Bangladeshi clinical psychologists • **Control:** None • No further information is provided.
Ventevogel et al. (8)	Afghanistan; Rural Eastern Nangarhar primary care facility	• **Participants:** General medical practitioners, nurses, Midwives, village health volunteers, Traditional birth attendants • **Intervention:** • Content: Doctors, nurses, and midwives were trained on general introduction to the basic concepts, principles of diagnosis and (psychopharmacological), and non-pharmacological treatment of mental health and mental illness, the effects of psychosocial problems on mental health, and to health education and health promotion • The village health volunteers and traditional birth attendants were trained on the basic knowledge of mental health and mental illness, detection of the mental problem related people and follow-up of patients with a chronic mental illness, based upon instructions of the doctors and nurses from the basic health centres • Duration: 2 months • Mood of delivery: Face-to-face through multimedia projector, series of handouts, video, group work • Delivered by: Mental health consultant • **Control:** None • No further information is provided
Jordan (66)	Sri Lanka; Colombo & Batticaloa tertiary care facility	• **Participants:** Mental health professionals, general medical practitioners, pastor, teacher-counsellor, health volunteer • **Intervention:** • Content: Basics of trauma assessment, family and community responses, reactions (acute stress disorder and PTSD), bereavement over the life cycle, threats of suicide, child and substance abuse; personal, pre-disposing, peri disposing, post-disposing, and preventive factors • Duration: Unavailable • Mood of delivery: Face-to-face through lectures, discussion, & role-play • Delivered by: Trained mental health volunteer • **Control:** None • No further information is provided

Most studies were conducted in rural primary care settings (*n* = 36) followed by tertiary care (*n* = 9), and other settings (*n* = 3). There were 31 quantitative studies (21 pre-post-studies, seven RCTs, two quasi-experimental design, and one case-control study). Five studies were qualitative and three mixed methods.

### Risk of Bias Within Studies

Six of the seven RCT studies were assessed as low-risk and one as high-risk. Among the non-randomised RCTs (*n* = 23), using the Robins-I tool, four studies were categorised as critical, 10 as serious risk, eight as moderate risk, and one as low risk of bias. We used MMAT to assess risk of bias for eight studies; four were categorised as low-risk, three as high-risk and one as critical risk of bias.

### Results of Individual Studies

#### Afghanistan (*n* = 1)

Tirmizi's et al. (24) quasi-experimental study reported results of training on depression delivered to primary healthcare workers in four districts in Afghanistan. The intervention group (*n* = 62) received recorded lectures and video training via smartphones and face-to-face lectures over 1 month with weekly Skype question and answer sessions. Details of the control group (*n* = 32) were not provided. There was a significant improvement post-test in the intervention group compared to the control (mean knowledge score = 16.06 vs. 6.80, *p* = 0.009).

#### Bangladesh (*n* = 1)

The WHO mhGAP-HIG (Humanitarian Intervention Guide) tool was evaluated by Tarannum et al. (25). Primary health care workers (*n* = 62) in Rohingya refugee camps were trained over 6-days to identify and manage people with mental, neurological, and substance use conditions. After seven months of training and supervision, researchers reported improvements in knowledge and skills (pre-post-test: 55 vs. 75%) and increased consultation rates from <40 to 160 per month.

#### India (*n* = 17)

Ashok Malla et al. (26) assessed in his part II paper, the effectiveness of mental health specialists training community health workers (*n* = 40) to provide basic mental health services for severe mental illnesses in an area of conflict. At 14 months, trained lay health workers diagnosed 262 new cases of severe mental disorders. Patients reported significant improvements in symptoms and functioning [*F* (*df* , 3.449) = 104.729, *p* < 0.001] and in all domains of quality of life including psychological health [*F* (*df* , 1.845) = 55.490, *p* < 0.001].

James et al. (27) evaluated the change in attitude of community health workers (Accredited Social Health Activists–ASHAs) toward people with mental illness after their involvement in a community-based rehabilitation program for people with severe mental illness. Community Attitudes to Mental Illness scale was administered at baseline and after 18 months of training and the study reported significant improvement in three of the four domains (*p* < 0.001).

Two studies by Maulik et al. (28, 67) evaluated the Systematic Medical Appraisal Referral and Treatment (SMART) Mental Health programme. The intervention comprised (1) an anti-stigma campaign, (2) training of ASHAs to screen for common mental disorders using Android tablets and to refer high-risk individuals to a primary care centre, (3) training of primary care doctors in WHO mhGAP-IG and to implement management guidelines using point-of-care decision support also using Android tablets, and (4) a system for ASHAs and doctors to follow-up patients. The 2017 study (67) evaluated a 10-day training delivered across 30 primary healthcare facilities in 15 villages with two primary care doctors and 21 ASHAs. ASHAs identified and referred 238 individuals with common mental disorders. Use of mental health services increased from 0.8% (*n* = 238) to 12.6% (*n* = 30) following the intervention. There was also a significant reduction in depression mean score (3.6, *p* < 0.0001) and anxiety mean score (1.3, *p* = 0.004). These results were built on in 2020 (28) among 40 ASHAs and five primary care doctors. Results showed increased mental health service use by patients from 3.3 to 81.2% (odds ratio 133.3, 95% CI 89.0–199.7; *P* < 0.001) and a significant reduction in mean depression and anxiety scores, from 13.4 to 3.1 (*P* < 0.001) and from 12.9 to 1.9 (*P* < 0.001) respectively.

Mehrotra's et al. (30) study examined Project ECHO; a hub and spoke tele-mentoring model that used video technology to connect mental health and addiction care specialists and healthcare providers in rural areas. Healthcare workers (*n* = 12) from rural hospitals received reading materials and participated in 12 fortnightly video conferences to discuss patients. Results showed improved knowledge (*t*-test: 3.71, *p* ≤ 0–01), self-confidence and efficacy (pre- vs. post-test: mean score: 58.16, SD = 12.23 vs. 70.83, SD = 11.06).

Another study by Muke et al. (29) studied the acceptability and feasibility of digital technology (mobiles, laptops, and tablets) to train ASHA workers (*n* = 32) on depression. They found it useful and convenient but recommended more interactive content, simple language, and greater use of images to increase engagement.

Two Indian studies focussed on older peoples' mental health. A qualitative study by Michael et al. (31) examined a geriatric mental health training program delivered to primary healthcare workers (*n* = 5). They found knowledge on depression, dementia, anxiety, substance abuse disorders, and use of medical and psychosocial interventions increased. Shaji et al. (40) evaluated dementia training undertaken by academic psychiatrists among 19 community health workers in Kerala using case vignettes. Community health workers identified 1,979 60-year-olds with suspected dementia. Health workers who received additional training identified a further 51 suspected cases; 33 confirmed dementia (positive predictive value of screening was 64.7%).

Shidhaye et al. (33) evaluated a 2-day training programme based on WHO mhGAP-IG training material and conducted under the Programme for Improving Mental health care (PRIME) district-level mental health care plan in rural India. The PRIME team carried out a separate 2-day training programme for 20 front-line workers and 15 paramedical staff on how to detect mental health within the community, where to refer, and how to provide mental health first aid. This was followed by weekly meetings between medical officers and the PRIME team. At 1-month follow-up, five patients were detected as depressed and three were referred to hospital. No pharmacological treatment was provided.

Patel et al. (38) assessed MANAS (MANAshanti Sudhar), a stepped-care intervention in Goa, involving a lay health counsellor, primary care physician, and visiting psychiatrist for individuals with depression and anxiety. Lay health counsellors received a 2-month structured training course to case manage patients, deliver non-drug treatments, and refer patients who do not get better to primary care physicians. Primary care physicians received half a day of training and a manual. Referral to a visiting psychiatrist was reserved for high-risk individuals or those who were unresponsive to earlier treatment. Adults who screened positive for a common mental disorder at primary care facilities (12 public, 12 private) were randomised to receive the MANAS intervention or “enhanced usual care” (*n* = 2,796) that consisted of primary care physicians who received only a tailored treatment manual. At public facilities, the MANAS intervention was consistently associated with strong beneficial effects over 12 months across all outcomes according to ICD-10 diagnoses: prevalence of common mental disorders (risk ratio = 0.70, 95% CI 0.53–0.92); depression (RR = 0.76, 95% CI 0.59–0.98); and suicide attempts and plans (RR = 0.64, 95% CI 0.42–0.98). In contrast, private facilities showed little evidence of impact.

Nimgaonkar's et al. (34) pre-post-test study evaluated a task-shifting programme in a rural tribal community. Health workers (*n* = 106) were trained to provide community education and identify and refer individuals with psychiatric problems to a community hospital. Psychiatric patients were followed up to improve treatment adherence. Results showed significant improvements in knowledge and attitude among participants (pre-post-test: 35 vs. 94%) and improvements in daily functioning among treated patients (pre-post-test: mean: 2.38 vs. 2.56; *p* = 0.01; No CI or SE) and self-referrals rates (27 vs. 57%) over 3 years. The cost of the program was USD 1.6 per person per annum.

Paudel et al. (35) examined the impact of depression and anxiety training delivered to community health workers (*n* = 26) by social workers. Focus group discussions found participants were able to identify symptoms of depression, suggest management options, and that they were more supportive and empathetic toward patients.

We found two studies that evaluated the effectiveness of Cognitive Behavioural Therapy (CBT) training in rural India. Beck et al. (36) evaluated a 5-day training programme for non-medical health professionals (*n* = 14) delivered by a clinical psychologist. Results on knowledge and clinical application of CBT were compared with UK participants of the same course; 9.2 vs. 9 and 18.7 vs. 16.3, respectively. Gupta and Aman (68) also evaluated change in knowledge among 20 psychologists, general medical practitioners, consultants, and medical students who received 3-day face-to-face training thorough role play and online supervision by psychiatrists. They found significant improvements in pre- and post- training knowledge among participants, *t*(13) = −6.899, (*p* ≤ 0.001, *r* = 0.886).

Armstrong's et al. (69) study investigated a 4-day mental health training program implemented among 66 community health workers. They were trained by two local healthcare professionals on mental health diagnosis, referrals, mental health first aid, and mental health promotion and were followed up after 3 months. Results showed an improved diagnostic capability in depression (pre-test: 22.7%, 95% CI = 13.3–24.7, *p* = 0.002) vs. post-test: 50.0%, 95% CI = 37.4–62.7, *p* = 0.002) and psychosis (pre-test: 9.1%, 95% CI = 3.4–18.7, *p* = 0.001) vs. post-test 27.3%, 95% CI = 10–39.6, *p* = 0.001) and a reduction in stigmatising attitudes among patients with mental illness (pre-test: 84.8% vs. post-test: 62.1%).

A study examining a training delivered by booklet to nurses (*n* = 35) at an Indian tertiary care hospital (37) on suicide prevention. They found improved knowledge (pre- and post-test: *Z* = 5.183, *p* ≤ 0.001) and attitude toward suicide attempted patients (pre- and post-test: *Z* = −4.380, *p* ≤ 0.001).

Thara et al. (39) conducted a retrospective study to determine the nature, quality and frequency of psychosocial support training delivered to community health workers after the 2004 tsunami in Tamilnadu. Participants (*n* = 127) were identified from government databases and included both government and NGO-employed community health workers and members of local women's voluntary groups. The majority reported to have benefited greatly from training, however, a lack of coordination led to overlap. Community members were generally happy with the interventions provided and were felt to be necessary for minimum 6 months.

#### Indonesia (*n* = 2)

In 2004, Indonesia integrated clinical psychologists into primary care and in 2015, an adapted WHO-mhGAP was incorporated as an add-on training to selected primary care doctors and nurses. Anjara's et al. (70) pragmatic two-arm cluster RCT investigated outcomes in patients who either received mental health care from doctors at primary care clinics who received WHO mhGAP training (*n*= 14 clinics) or co-located clinical psychologists (*n* = 14 clinics). Outcomes of doctor care (*n* = 153 patients) were proven to be statistically not inferior to clinical psychologists (*n* = 141 patients) in reducing symptoms of social and physical impairment, reducing disability, and improving health-related quality of life at 6 months. Economic analyses indicate lower costs and better outcomes in the co-located clinical psychologist arm.

Citraningtyas et al. (41) conducted a mixed method evaluation of a 5-day Capacity Building for Child and Adolescent Mental Health in Disaster Areas (CAMHD) training-of-trainers in Jakarta. Participants (*n* = 16) were non-specialist resident doctors, psychiatrists, psychologists, and social workers. Pre-post-test results showed a significant improvement of knowledge (mean = 2.75; 13.75%; *p* < 0.001) among participants. In qualitative interviews, participants identified the benefits of learning using case studies and role play.

#### Nepal (*n* = 11)

Acharya's et al. (50) evaluated a WHO mhGAP-based training delivered digitally and on-site. Clinicians (*n* = 27) at a rural Nepali primary care centre viewed five video lectures followed by on-site skills-building and clinical coaching to help use new knowledge in clinical practise. Pre-post-tests indicated a significant increase in median scores on knowledge questionnaires for acute stress (median increase: 20, *p* = 0.03), grief (40, *p* < 0.01), psychosis (22, *p* = 0.01), and post-traumatic stress disorder (PTSD) (20, *p*= 0.01) but not for depression (11, *p* = 0.12).

Two RCT studies evaluated the effect of training community health workers to deliver psychosocial interventions (47, 48). Markkula et al. (48) evaluated a 5-day training programme in psychosocial counselling, basic therapeutic skills, CBT, problem-solving, exposure therapy, yoga, and meditation. The control group received 5-day training on detecting and treating mental health disorders with a 3-day refresher course (enhanced usual care). A total of 287 participants (*n* = 141 intervention; *n* = 146 control arm), predominantly socially disadvantaged women, were randomised to each arm. The researchers found a significant reduction in depression measured by Beck Depression Inventory (BDI) scale [mean difference −7.43 (95% CI −9.71 to −5.14)] and anxiety (mean difference −5.42, 95% CI −7.59 to −3.27) at 6 months in the intervention compared to the control group suggesting lay psychosocial counselling is effective in treating depression and anxiety.

Sangraula et al. (47) evaluated the training of psychosocial health workers by an NGO to deliver Problem Management Plus (PM+), a five-session intervention designed for individuals with psychological distress living in communities affected by adversity. People diagnosed with depression were randomly allocated to receive PM+ (*n* = 61) or enhanced usual care (*n* = 60) which consisted of family meetings, psychosocial support, and information on local mental health services. Patients with mental illness who received PM+ showed a significantly greater reduction in depression (mean score = −3.5), common psychological problems (mean score = −1.0), PTSD (mean score = −2.7) and an increase in perceived social support (mean score = 0.9) compared to the control group.

We found six studies that focussed on examining the impact of the PRIME district mental health plan, an intervention to scale up treatment for alcohol use disorder, depression, epilepsy, and psychosis in primary care in Chitwan, Nepal. As part of PRIME, primary care workers were trained in the WHO mhGAP. Kohrt et al. (49) conducted an evaluation of 44 primary care workers who took part in the training. They found improved mean scores for knowledge (10.37, *p* < 0.001, *d* = 1.75) and competencies (7.87, *p* < 0.001, *d* = 1.44) on mental health and reduced stigma toward people with mental illness (−3.25, *p* = 0.003, *d* = −0.55).

As part of PRIME, community health workers were trained to use the Community Informant Detection Tool (CIDT) tool to identify and refer individuals with mental illness to primary care centres. Jordans et al. conducted two studies (44, 51) to evaluate this training. In their pragmatic RCT, community health workers at 24 health facilities were randomly assigned to receive “standard and CIDT training” (intervention) or “standard” training alone (control). All participants were trained on mental health, stigma, mental health services in primary care, and self-referral. The CIDT group received additional training in proactive community referrals consisting of vignettes and pictures designed for low literacy populations. The authors compared the number of patients registered at health facilities before and 6 months post training; 309 patients were registered at health facilities in the CIDT group compared with 182 patients in the control group (median = 24 vs. 16, *p* = 0.04, *r* = 0.42).

Aldridge et al. (42) evaluated the indirect impact of PRIME on suicidal ideation among individuals diagnosed with depression (*n* = 137 treatment; *n* = 75 control) and alcohol use disorder (AUD) (*n* = 175 treatment; *n* = 57 comparison). Participants in the treatment group received psychotropic medication, psychosocial support, and psychoeducation according to mhGAP-IG clinical decision algorithm. The control group received standard primary care with no mental health treatment provided. Patients with depression in the treatment group had a greater reduction in ideation compared to the control group at 12 months (OR = 0.31, 95% CI: 0.08–1.12; *p* = 0.07), with a significant treatment over time effect (*p* = 0.02). Among the AUD group, there was no significant difference in suicidal ideation between treatment and control group at 12 months (OR = 0.46, 95% CI: 0.06–3.27; *p* = 0.44), and no treatment over time effect (*p* = 0.72).

Kohrt et al. (45), conducted a mixed-method evaluation of integrating anti-stigma modules (entitled RESHAPE) into the PRIME mental health care program. The adapted training curriculum was delivered in two sessions to 41 primary healthcare providers. In qualitative interviews, primary healthcare providers described positive changes in their views that individuals with mental illness are violent and their ability to treat mental illness effectively. At 16 months, their willingness to interact with individuals with a mental illness increased from 54% pre-training to 81% and observed clinical competency increased from 49 to 93%.

Luitel et al. (46), conducted a process evaluation of the implementation of PRIME in Chitwan. They reported challenges due to low levels of mental health literacy, heavy workload among primary care workers, frequent transfer of trained staff, stigma, inadequate physical facilities, lack of private consultation rooms, complex and lengthy drug procurement and distribution processes, and poor mental health supervision in primary care. They found primary care workers were motivated to learn about mental healthcare, and that mentoring and supervision provided by psychiatrists, support from the Ministry of Health and the District Public Health Office, and procurement of new psychotropic medicines helped overcome implementation challenges.

#### Pakistan (*n* = 5)

Three studies evaluated the “Thinking Healthy Program” (THP), a CBT-based training delivered to community health workers to treat perinatal depression that has been integrated into the heath system in Pakistan. Rahman et al. (71) evaluated a digital adaptation of THP. The Technology-Assisted Cascaded Training and Supervision (TACTS) system facilitated the training of community health workers using an Android tablet through a cascade training/supervision model. The cascade model began with a specialist THP master trainer (*n* = 1) training a non-specialist THP trainer (*n* = 1; psychology graduate) in a 5-day workshop and a Skype supervision session. These non-specialist trainers then delivered a 5-day technology-assisted training to four community health workers trained as supervisors and continued to supervise them via monthly Skype sessions. These supervisors then cascaded the 5-day training to 40 community health workers. Eighty community health workers were randomly assigned to either TACTS or conventional face-to-face training and supervision by a specialist. Results indicated no significant differences between health workers trained using TACTS and supervised from distance vs. those trained and supervised by a specialist face-to-face (mean ENACT score 24.97 vs. 27.27, *p* = 0.079, 95% CI 4.87–0.27) and at 3 months follow-up assessment (44.48 vs. M = 43.63, *p* = 0.53, CI −1.88–3.59).

Zafar et al. (54) qualitative study evaluated THP training for 40 community health workers within a combined nutrition and early child development program was found to be useful and effective. THP training was conducted in the first 2 days of a 5-day training program, and skills learned were repeatedly practised throughout the five days. Study participants were able to apply these skills effectively and the approach was found to be useful by community health workers, mothers, and their families.

Rahman's et al. (52) RCT examined the THP training which was delivered to community health workers using a manual, which could be kept for reference. Mothers in the intervention clusters (*n* = 463) received the THP training from 20 specially trained community health workers. Mothers in the control clusters (*n* = 440) received the same number of visits to those in the intervention group but by routinely trained community health workers (*n* = 20). At 6 months, 23 and 53% mothers in the intervention and control groups, respectively, met the criteria for major depression [adjusted odds ratio (OR) 0.22, 95% CI 0.14–0.36, *p* < 0.0001]. These effects were sustained at 12 months (27 vs. 59%, adjusted OR 0.23, 95% CI 0.15–0.36, *p* < 0.0001).

Humayun et al. (53) evaluated a mhGAP training delivered to 56 physicians and psychosocial staff in a conflict-affected district in Pakistan. The 2-day training was delivered by psychiatrists and focused on diagnosis, treatment, follow-up, and referral of mental, neurological and substance use disorders. Participant mean pre- and post-test scores were 15.43, 62% (*p* = 0.000, S.D. 4.05) and 19.48, 78% (*p* =0.000, S.D. 3.13), respectively, which showed significant improvement.

Taj et al. (55) conducted a 5-day face-to-face multi-method teaching program for 50 mental health professional on practical skills to manage depression, anxiety, substance abuse, and other common problems through role plays and video and slide presentations by mental health professionals. Result showed an improved knowledge score (pre- vs. post-test: 65 vs. 74%).

#### Sri Lanka (*n* = 3)

We found two studies evaluating training that used WHO mhGAP guidelines to integrate mental health into primary care in Sri Lanka. Siriwardhana et al. (56) found knowledge scores improved after a 3-day training delivered to primary care practitioners (*n* = 12) serving post-conflict populations (pre- vs. post-test: 72.8%, SD: 11.4 vs. 77.2%, SD: 11.8). Jenkins et al. (58) evaluated the training of 260 primary care staff in five areas in Sri Lanka and found improved knowledge in pre- vs. post-test scores in all.

Blignault et al. (57) conducted a two phased collaborative capacity-building training program adopting a train-the trainer model. In phase I, 14-day face-to-face training course took place in Sydney, Australia for eight senior clinicians to improve their identification and management of mood disorders. After 3 months, they received 5-day follow-up training and went on to train 48 medical officers of mental health and 42 psychiatric support workers and general counsellors over 5 days (phase II). Results demonstrated the improved mental health knowledge (pre- vs. post-test: 4.5 ± 2.5 vs. 6.2 ± 2.0, CI = 0.86–2.71, *p* = 0.0002) and attitude toward people with mental illness (pre- vs. post-test: 36.9 ± 6.9 vs. 32.4 ± 7.2, CI = 2.82–6.15, *p* = 0.0000) among participants.

### Synthesis of Results

Among 48 reviewed articles, training was provided and evaluated in 39 studies (81%) (24–31, 33–58, 67–71) (Table 2) and another nine studies (19%) (42–51) (Table 3) only described the intervention or training module and did not report any results.

Of the 39 studies, the majority focused training to general medical practitioners (*n* = 13, 33%) (24, 28, 34, 38, 41, 50, 53, 56–58, 67, 68, 70) followed by nurses (*n* = 5, 13%) (25, 34, 37, 45, 70), psychologists (*n* = 4, 10%) (25, 30, 41, 68), psychiatrists (*n* = 2, 5%) (41, 58) and other non-clinical professionals such as community healthcare workers (*n* = 29, 74%) (24, 26–31, 33–35, 38–54, 57, 67), health or medical assistants (*n* = 4, 10%) (25, 45, 50, 69), health educators (*n* = 2, 5%) (25, 71) and paramedics (*n* = 1, 3%) (33). Thirty-two studies (82%) were conducted in primary healthcare facilities (24, 26–31, 33–36, 38, 39, 41–44, 46–48, 50, 52–58, 67, 69–71) followed by five studies (13%) in tertiary care settings (37, 40, 45, 49, 51), and two studies (5%) in refugee camps or other (25, 68).

Twenty-three (59%) studies provided interventions that addressed common mental disorders (CMD) (24, 25, 29, 34–36, 38, 39, 41–43, 47–49, 52, 54, 55, 57, 67–71), five (13%) addressed severe mental illness (26, 27, 30, 37, 40), and 10 studies (26%) addressed both common and severe illnesses (28, 31, 33, 44, 45, 49, 50, 53, 56, 58). Thirty-six studies (92%) provided training to participants face-to-face (24–28, 31, 33–58, 67–70) and eight (21%) used digital technology and web-based training (24, 28–30, 50, 67, 68, 71). Training programmes were delivered in a variety of formats, such as lectures and slide presentations, role play, case discussions, audio-visual material, question and answer sessions, team review sessions, and workshops. Most of the training sessions were discrete and they ranged from one to 30 days.

Training interventions were commonly delivered by multi-disciplinary teams (*n* = 12, 31%) comprising of psychiatrists, psychologists, counsellors, and social workers (25, 26, 28, 30, 33, 37, 41, 49, 56, 67, 69, 70). Six studies (15%) were delivered by a psychiatrist (27, 32, 34, 40, 50, 53), two (5%) by a psychologist (36, 71), and one (3%) by a social worker (35). Eighteen studies did not provide any information regarding trainers. The content of interventions were highly heterogenous. Twelve (31%) studies adapted WHO mhGAP guidelines (24, 25, 28, 33, 42, 44–46, 49, 53, 56, 70) of which six studies (15%) were conducted under the auspices of the PRIME program (33, 42, 44–46, 49) and one integrated the RESHAPE module (3%) (45). Three studies (5%) conducted the THP training (52, 54, 71), two studies (5%) delivered the SMART module (28, 67). There was one study conducted for PM+ training (3%) (47), the MANAS trial (3%) (38) and the SPRING program (3%) (54).

Thirteen (33%) studies trained participants to deliver basics of mental health treatment including eight (21%) studies trained healthcare workers to deliver psychotherapy like CBT (36, 39, 40, 48, 52, 54, 68, 71), psychoeducation, psycho-social counselling, pharmacotherapy, behavioural activation therapy, meditation, and yoga (27, 29, 35, 43, 69).

Twenty-three studies (59%) evaluated the knowledge, skills and attitude of healthcare workers (24, 25, 29–31, 33, 35–37, 41, 43, 45, 46, 49, 50, 53–58, 68, 69), 18 studies (46%) assessed diagnostic capabilities and competencies (26, 27, 29, 30, 33–35, 40–43, 45–47, 51, 54, 69, 71) three (8%) studies assessed referral ability (35, 43, 51), four (10%) measured stigma among healthcare workers (26, 46, 49, 69) and three (8%) assessed patient's well-being status (43, 47, 51).

The mental health status of individuals who received care from trained healthcare workers was measured using a range of scales, including WHODAS (WHO Disability Assessment Schedule), GHQ-12 (General Health Questionnaire-12), PHQ-9 (Patient Health Questionnaire-9), GAD-7 (Generalised Anxiety Disorder), MICA (Mental Illness: Clinicians' Attitudes), and ENACT (Enhancing Assessment of Common Therapeutic factors).

#### Enabling Factors and Barriers

We found digital technology was an effective enabling factor in implementing training among healthcare workers in primary and community settings. Large-scale interventions found it was important to have early and sustained involvement of key stakeholders, including traditional healers and religious leaders. Training that involved a higher number of sessions, a multidisciplinary team, and where there was longer term supervision and mentoring, typically had sustained effects. Other enabling factors included using simple language, synonyms for difficult words, contextually specific images, and interactive content. Interventions that were of low cost and did not require a large time commitment had good participation rates.

Barriers to implementation included the lack of physical space to deliver the training program, as well as implementing the training itself, for example a separate room to offer psychosocial counselling. Other studies reported practical issues such as transport for healthcare workers to attend, inadequate numbers of trainers, and a lack of collaboration between different training providers to adhere to time constraints of the program. Several studies mentioned the quality of training arrangements, such as smaller numbers of sessions and difficulty in understanding guidelines as critical barriers for healthcare workers. Additionally, issues of stigma and inadequate mental health knowledge affected participation rates. Two studies mentioned healthcare workers were re-assigned or retired and lost to follow up.

A few studies described how patients themselves were unwilling to receive treatment due to issues of stigma or as a result of low mental health literacy. A lack of essential psychotropic medications and adequate transport to the healthcare facility were further barriers for patients seeking mental health care.

#### Cost Effectiveness

Only one study conducted a cost-effectiveness analysis (71). In this study, two types of interventions such as technology-assisted cascaded training and supervision (TACTS) and conventional face-to-face training were provided to 40 community healthcare workers. Results showed that costing of a TACTS training program was equally effective and 30% less expensive than the conventional methods of training and supervision.

### Risk of Bias Across the Studies (*n* = 39)

We examined the risk of bias in 39 of the 48 studies. Thirty-one were quantitative study designs, five qualitative and three mixed methods. Twenty-one quantitative studies had a pre-post-design, of which 10 were appraised with high risk of bias (25, 43, 49, 51, 55–57, 67–69), followed by moderate (*n* = 6) (26, 27, 30, 34, 40, 53), critical (*n* = 4) (36, 37, 50, 58) and low risk (*n* = 1) (45). Two quasi-experimental studies (24, 28) and one case-control studies (42) were assessed as moderate risk of bias.

We assessed seven RCT studies using Cochrane Collaboration tool; six were low risk of bias (*n* = 6) (38, 44, 47, 48, 52, 71) and only one study was assessed high risk (70). Qualitative studies were appraised using MMAT; three were assessed as low risk of bias (29, 35, 39), one high (54) and one critical risk (31). Among three mixed method studies two studies were low risk (41) and two were high risk of bias (33, 46).

### Additional Analysis

Additional analysis such as meta-analysis was not conducted due to the extreme heterogeneities in terms of population, intervention contents, modalities, measurement scales, and outcome of those studies.

## Discussion

### Summary of Evidence

We identified 48 studies since 2000 that focussed on mental health training and upskilling health care workers from South and South-East Asia. Most studies were concentrated in India or Nepal and two studies were conducted in South-East Asia. It is unclear why so few were conducted in South-East Asia, as both regions face a similar mental health burden, experience insufficient levels of investment, and a paucity of mental health policies.

Most of the focus of mental health training was directed toward improving primary healthcare workers' ability to identify, manage, and refer people with mental illness. This finding is consistent with studies in other developing countries (72–75) as well as research from high income countries with stronger health care systems that report intervening in primary care is effective (76, 77). Primary healthcare facilities in countries such as Bangladesh, Pakistan and India can serve between 6,000 and 30,000 people (78–80). Therefore, sensitising primary level health workers about mental health is an efficient use of scarce resources (81, 82). Each country had varied types of primary healthcare workers with differing knowledge and skill gaps; this should be acknowledged when designing interventions (74, 75).

Our review findings are consistent with task sharing research published in other LMICs (83–86) that suggest that community health workers, who have limited or no prior training in the mental health, can effectively deliver psychological interventions. Scaling up these training programmes has potential to strengthen the wider health system as effective, yet the least resource intensive treatment, can be delivered to patients first; only “stepping up” to intensive/specialist services as clinically required (38, 87, 88).

While there was high heterogeneity on the content of interventions, many utilised established mental health modules such as the WHO mhGAP guidelines. Overall, these modules were successful in increasing mental health knowledge and improving the treatment and management of people with mental ill-health (89). Training programmes such as the THP and PM+ demonstrated the broader role of the mhGAP-IG in encouraging the development and evaluation of acceptable, feasible and scalable talking therapies in the South and South-East Asian region. Further evidence on the process of how interventions are being tailored would benefit future efforts to scale established mental health training modules (90, 91).

Many successful interventions were provided by multidisciplinary teams that consisted of both specialists (such as psychiatrists and psychologists) and non-specialists (such as community health workers). Existing research suggests this is an essential tool for constructing an effective and patient-centred healthcare delivery system (92, 93) as it can break down the hierarchy and power of individuals, give more leverage to workers, and produce a higher level of work and job satisfaction (92, 94, 95).

Most interventions were delivered face-to-face, and participants valued content delivered in an interactive manner, for example through role play, case discussion, and question and answer sessions. More recent studies were successful in leveraging digital technology to deliver training and provide ongoing virtual supervision to rural and remote areas and areas with scarce resources. This is consistent with research supporting the benefits of online education and training to overcome geographical, human resource and cost barriers without sacrificing on knowledge outcomes (96, 97). However, it is important to select appropriate digital devices and applications for delivering the intended content, and that these interventions can function offline or within network connectivity constraints of the target setting.

We found only a few studies (26, 28, 34, 38, 42, 47, 48, 51, 52, 67, 69, 70) that evaluated the impact of training in terms of patient outcome. Most studies focused on the initial three stages (reaction, learning, and behaviour) developed by Kirkpatrick and Kirkpatrick (98) in evaluating impact of training. Future studies need to evaluate the effect of training on patient outcome as well as participants.

### Limitations

In many LMICs, volunteers trained by NGOs can play a critical role in the provision of mental healthcare. However, they are excluded in this review as their role in the healthcare workforce is not a sustainable solution for health systems to achieve universal health coverage. Mainstream health services require capacity for basic provision of mental healthcare. We only included studies published in English and may have missed valuable evidence published in the native language to the country where the study was conducted. A broad range of countries were included with diverse health systems and differential need, and this may limit how we generalise our findings to the whole region.

### Implications for Policy Makers and Researchers

We found few studies that offered supervision and support post-training. Training programmes should ensure that there is continued opportunities for refresher training and/or supervision of healthcare workers as a critical component to ensuring quality of care. Future research should assess long term outcomes in patients to determine if application of newly acquired knowledge and skills in everyday practise is sustained.

Most interventions were designed to detect and manage common mental disorders with severe mental illnesses, such as schizophrenia, severe depression and bipolar affective disorder, less frequently targeted. It is important that healthcare workers can identify and manage individuals with severe mental illnesses, particularly in light of the high burden in the region. Additionally, few studies targeted the upskilling of existing mental health specialists. Even if task-shifting to community health workers is implemented extensively, the need for mental health specialists will continue as they play an essential role in delivery of services and in training, supervision, and mentoring of non-specialist workers (99).

We found only one study that assessed cost effectiveness of intervention (71). Less than 1% of national budgets in the South and South-East Asian region is allocated to mental health, and so the mobilisation of financial resources to develop the healthcare workforce is a major challenge to strengthening mental health systems. It is therefore critical that rigorous cost-effectiveness studies are conducted to inform the design and scaling-up of training interventions.

## Conclusions

Training primary and community health workers in the identification and treatment of mental health disorders can lead to significant improvements in knowledge and to the effective delivery of mental health care, through community-based programmes and task-shifting approaches. Future research in the field should focus on severe mental illnesses, upskilling healthcare workers along the entire care pathway, and examining the sustainability of knowledge and skills gained and the role of ongoing supervision.

## Data Availability Statement

The original contributions presented in the study are included in the article/Supplementary Material, further inquiries can be directed to the corresponding author/s.

## Author Contributions

KK, CB, IK, and SG developed the study protocol with the contributions from MR, GL, and SR and registered in PROSPERO. KK, CB, IK, and SG developed the search strategy and searched the articles with the help of librarian. Titles, abstracts and full texts of the retrieved articles were screened separately by KK and CB. SG resolved any conflicts as a third reviewer. KK, CB, and IK conducted the data extraction and narrative synthesis with the guidance of SG. KK, CB, IK, and SG developed the manuscript. All authors contributed, critically provided feedback and approved the final version of the manuscript for submission.

## Funding

This project was funded and supported by the United Kingdom Research and Innovation (UKRI) Global Challenges Research Fund (GCRF) (Grant Reference Number WPPA-G24119).

## Author Disclaimer

The views expressed are those of the author(s) and not necessarily those of the UKRI.

## Conflict of Interest

The authors declare that the research was conducted in the absence of any commercial or financial relationships that could be construed as a potential conflict of interest.

## Publisher's Note

All claims expressed in this article are solely those of the authors and do not necessarily represent those of their affiliated organizations, or those of the publisher, the editors and the reviewers. Any product that may be evaluated in this article, or claim that may be made by its manufacturer, is not guaranteed or endorsed by the publisher.
