# Supplementary material for: Educational and Training Interventions Aimed at Healthcare Workers in the Detection and Management of People With Mental Health Conditions in South and South-East Asia: A Systematic Review
Source: Front Psychiatry. 2021 Oct 11;12:741328. doi: 10.3389/fpsyt.2021.741328 (PMC8542900; doi:10.3389/fpsyt.2021.741328)
Supplement: Supplementary file 1 [file Table_1.DOCX]

**Supplementary Table 1: Search strategy**

| **Medline:**  **1. Population**  **Mesh terms:**  exp health personnel/  **Key words:**  (psychologist* or psychiatrist* or nurse* or doctor* or physician* or general practi* or hospitalist* or clinician* or close to community provider).mp. ((lay or health) adj counsellor).mp. (medical adj (practi* or staff or personnel or profession*)).mp. (healthcare adj2 (worker* or provider* or personnel or professional*)).mp. (Health* adj2 (worker* or workforce or Personnel or professional*)).mp. (primary health adj2 (worker* or provider* or practitioner*)).mp. (community adj2 (worker* or health care provider* or health worker* or healthcare worker* or primary care provider* or primary healthcare provider* or health officer* or health promoter* or health volunteer* or health assistant* or volunteer*)).mp. (Physician Assistants or Medical assistant or Clinical officer or Emergency Medical Technicians or Primary care paramedic or Advanced care paramedic).mp. (lay adj2 (worker or health worker or health counsellor or counsellor)).mp.  **2. Intervention**  **Mesh terms:**  exp education/ exp teaching/ capacity building/  **Key words:**  **Capacity build***  (train* or teach* or educate or upskill* or instruct* or capacity build*).mp. ((career or staff or personal or profession* or personnel) adj2 development).mp.  **3. Context**  **Mesh terms:**  bangladesh/ or bhutan/ or exp india/ or afghanistan/ or nepal/ or pakistan/ or sri lanka/ or thailand/ or myanmar/ or North Korea/ or indonesia/ or Timor-leste/  **Key words:**  (South$ or west*) asia*.mp. Indian subcontinent.mp. Indian ocean islands.mp. bangladesh* or bhutan* or nepal* or india* or afghan* or pakistan* or sri lanka* or maldiv* or Thai* or Thailand or Myanmar or Burmese or North korea or Democratic People's Republic of Korea or Indonesia* or Timor-Leste or Timorese).mp.  **4. Mental health context**  **Mesh terms:**  Exp psychological stress/ or exp mental health/ or exp mental disorders/ or exp affective disorders/ or exp psychological stress/ or exp depression/ or exp anxiety/  **Key words:**  (depress* or anxi* or mood disorder).mp. suicid* (mental adj2 (health or stress or disorder* or illness* or problem*)).mp. (psychological adj2 (disorder* or illness* or problem*)).mp. (Psychiatric adj1 (disorder* or illness* or problem*)).mp.  **Total (1+2+3+4)** | **1,650,693**  **1,391,719**  **298,472**  **1,903,933**  **998** |
| --- | --- |
| **Embase:**   1. **Population**   **MeSh terms:**  exp health care personnel/ or exp advanced practice provider/ or exp care coordinator/ or exp clinician/ or exp emergency medical dispatcher/ or exp health auxiliary/ or exp health educator/ or exp health workforce/ or exp hospital personnel/ or exp lay health worker/ or exp medical personnel/ or exp mental health care personnel/ or exp nursing home personnel/  **Key words:**  (psychologist* or psychiatrist* or nurse* or doctor* or physician* or general practi* or allied health personnel or clinician*).mp. ((lay or health) adj counsellor).mp. (medical adj (practi* or staff or personnel or profession*)).mp. (health* care adj2 (worker or provider or personnel or professional)).mp. (Health adj2 (Workforce or Personnel or professional)).mp. (primary health care adj (worker or provider or practitioner)).mp. (community adj (worker* or health care provider* or health worker* or healthcare worker* or primary care provider* or primary healthcare provider* or health officer or health promoter or health volunteer* or health assistant* or volunteer)).mp. (Physician Assistants or Medical assistant or Clinical officer or Emergency Medical Technicians or Primary care paramedic or Advanced care paramedic).mp. (lay adj2 (worker or health worker or health counsellor or counsellor)).mp.   1. **Intervention**   **Mesh terms:**  exp education/ or exp continuing education/ or exp education program/ or exp educational technology/ or exp health education/ or exp in service training/ or exp learning environment/ or exp medical education/ or exp problem based learning/ or exp teaching/ or exp vocational education/ exp teaching hospital/ or exp teaching/ exp capacity building/  **Key words:**  (train* or teach* or educate or upskill* or instruct or capacity build*).mp. ((career or staff or personal or profession* or personnel) adj development).mp.   1. **Context**   **Mesh terms:**  Exp south asia/  **Key words:**  South$ asia*.mp.  Indian subcontinent.mp. bangladesh* or bhutan* or nepal* or india* or afghan* or pakistan* or sri lanka* or maldiv* or Thai* or Thailand or Myanmar or Burmese or North korea or Democratic People's Republic of Korea or Indonesia* or Timor-Leste or Timorese).mp.   1. **Mental health context**   **Mesh terms:**  exp depression/ or exp stress, psychological/ or exp anxiety disorders/ or exp mood disorders/ or mental disease/ or mental health/  **key words:**  (depress* or anxi* or mood disorder).mp. suicid* (mental adj2 (health or stress or disorder* or illness* or problem*)).mp. (psychological adj2 (disorder* or illness* or problem*)).mp. (Psychiatric adj1 (disorder* or illness* or problem*)).mp.  **Total (1+2+3+4)** | **2,774,248**  **2,289,739**  **505,992**  **1,674,112**  **2348** |
| **Psychinfo**   1. **Population**   **Mesh terms:**  exp health personnel/ or exp professional personnel/ or exp allied health personnel/ or exp caregivers/ or exp medical personnel/ or exp mental health personnel/ or exp counselors/ or exp home care personnel/ or exp professional measures/ or exp rescue workers/ or exp social workers/ or exp teleconsultation/ or exp therapists/  **Key words:**  (psychologist* or psychiatrist* or nurse* or doctor* or physician* or general practi* or allied health personnel or clinician*).mp. ((lay or health) adj counsellor).mp. (medical adj (practi* or staff or personnel or profession*)).mp. (health* care adj2 (worker or provider or personnel or professional)).mp. (Health adj2 (Workforce or Personnel or professional)).mp. (primary health care adj (worker or provider or practitioner)).mp. (community adj (worker* or health care provider* or health worker* or healthcare worker* or primary care provider* or primary healthcare provider* or health officer or health promoter or health volunteer* or health assistant* or volunteer)).mp. (Physician Assistants or Medical assistant or Clinical officer or Emergency Medical Technicians or Primary care paramedic or Advanced care paramedic).mp. (lay adj2 (worker or health worker or health counsellor or counsellor)).mp.   1. **Intervention**   **Mesh terms:** exp Continuing Education/ or exp Individual Education Programs/ or exp Career Education/ or exp Adult Education/ or exp Psychology Education/ or exp "Accreditation (Education Personnel)"/ or exp Distance Education/ or exp Counselor Education/ or exp Health Education/ or exp Graduate Education/ or exp Business Education/ or exp Graduate Psychology Education/ or exp Medical Education/ or exp Art Education/ or exp Nursing Education/ or exp Cooperative Education/ or exp Multicultural Education/ or exp Education/  **Key words:**  Capacity build*.mp. (train* or teach* or educate or upskill* or instruct).mp. ((career or staff or personal or profession* or personnel) adj development).mp.   1. **Context**   **Key words:** (bangladesh* or bhutan* or nepal* or india* or afghan* or pakistan* or sri lanka* or maldiv* or Myanmar or Burmese or Thailand or Thai* or North Korea* or Bhutan* or Indonesia* or Timor-Leste or Timorese).mp. Indian ocean islands.mp. Indian subcontinent.mp. South$ asia*.mp.   1. **Mental health context**   **Mesh terms:**  exp Psychological Stress/ or exp Mental Health/ or exp mental disorders/ or mental health/ or exp affective disorders/  **Key words:**  (depress* or anxi* or mood disorder).mp. suicid* (mental adj2 (health or stress or disorder* or illness* or problem*)).mp. (psychological adj2 (disorder* or illness* or problem*)).mp. (Psychiatric adj1 (disorder* or illness* or problem*)).mp.  **Total (1+2+3+4)** | **663,829**  **912,401**  **65,588**  **1,351,808**  **840** |
| **Global health**   1. **Population**   **Mesh terms:**  exp personnel/ or hospital personnel/ or exp medical auxiliaries/  **Key words:**  (psychologist* or psychiatrist* or nurse* or doctor* or physician* or general practi* or allied health personnel or clinician*).mp. ((lay or health) adj counsellor).mp. (medical adj (practi* or staff or personnel or profession*)).mp. (health* care adj2 (worker or provider or personnel or professional)).mp. (Health adj2 (Workforce or Personnel or professional)).mp. (primary health care adj (worker or provider or practitioner)).mp. (community adj (worker* or health care provider* or health worker* or healthcare worker* or primary care provider* or primary healthcare provider* or health officer or health promoter or health volunteer* or health assistant* or volunteer)).mp. (Physician Assistants or Medical assistant or Clinical officer or Emergency Medical Technicians or Primary care paramedic or Advanced care paramedic).mp. (lay adj2 (worker or health worker or health counsellor or counsellor)).mp.   1. **Intervention**   **MeSh terms:** exp professional education/ or exp education/ or exp career education/ or exp continuing education/ or exp education programmes/ or exp medical education/ or exp community education/ or exp adult education/ or exp non-formal education/ or exp practical education/ or exp competency-based education/  exp teaching/  **Key words:**  Capacity build*.mp. (train* or teach* or educate or upskill* or instruct).mp. ((career or staff or personal or profession* or personnel) adj development).mp.   1. **Context:**   **Mesh terns:**  exp south asia/  **Key words:** (bangladesh* or bhutan* or nepal* or india* or afghan* or pakistan* or sri lanka* or maldiv* or Myanmar or Burmese or Thailand or Thai* or North Korea* or Bhutan* or Indonesia* or Timor-Leste or Timorese).mp. Indian ocean islands.mp. Indian subcontinent.mp. South$ asia*.mp.   1. **Mental health context**   **Mesh terms:**  exp depression/ or exp mental health/ or exp anxiety/  **Key words:**  (depress* or anxi* or mood disorder).mp. suicid* (mental adj2 (health or stress or disorder* or illness* or problem*)).mp. (psychological adj2 (disorder* or illness* or problem*)).mp. (Psychiatric adj1 (disorder* or illness* or problem*)).mp.  **Total (1+2+3+4)** | **166,178**  **166,302**  **214,290**  **104,134**  **218** |
| **CINAHL**   1. **Population**   psychologist* OR psychiatrist* OR doctor* OR physician* OR (general AND practition*) OR clinician* OR (allied AND health AND personnel) OR nurse* OR ((medical) AND (practition* OR staff OR personnel OR profession*)) OR ((health* AND care) AND (worker OR provider OR personnel OR professional)) OR ((health) AND ((workforce OR personnel OR professional)) OR ((primary AND health AND care) AND (worker OR provider OR practitioner)) OR ((community) AND (worker* OR (health AND care AND provider*)) OR (health AND worker*) OR (healthcare AND worker*) OR (primary AND care provider*) OR (primary AND healthcare AND provider*) OR (health AND officer) OR (health AND promoter) OR (health AND volunteer*) OR (health AND assistant*) OR (physician AND assistant*) OR (medical AND assistant*) OR (clinical AND officer*) OR (medical AND technician*) OR paramedic* OR ((lay) AND (worker OR (health AND worker))) OR (health AND counsellor) OR counsellor OR ((lay OR health) AND counsellor)   1. **Intervention**   train* OR teach* OR educate OR upskill* OR instruct OR ((career OR staff OR profession* OR personal OR personnel) AND (development)) OR (capacity AND build*)   1. **Context**   ((south* or west* AND asia*)) OR bangladesh* OR India* OR bhutan* OR nepal* OR pakistan* OR (sri lanka*) OR maldiv* OR afghan* OR Myanmar OR Burmese OR Thai* or North Korea* or Bhutan* or Indonesia* or Timor-Leste or Timorese)   1. **Mental health context**   anxi* OR depress* OR (mood AND disorder) OR (mental AND health) OR (mental AND disorder) OR (mental AND illness) or suicid* OR (psychiatric AND disorder) OR (psychological AND disorder*) or (psychological AND illness*) OR (psychological AND problem*) OR (psychiatric AND disorder*) OR (psychiatric AND illness*) OR (psychiatric AND problem*)  **Total (1+2+3+4)** | **328,358** **1,880,498** **113,551** **486,502**  **399** |
| **Cochrane**   1. **Population**   psychologist* OR psychiatrist* OR doctor* OR physician* OR (general AND practition*) OR clinician* OR (allied AND health AND personnel) OR nurse* OR ((medical) AND (practition* OR staff OR personnel OR profession*)) OR ((health* AND care) AND (worker OR provider OR personnel OR professional)) OR ((health) AND ((workforce OR personnel OR professional)) OR ((primary AND health AND care) AND (worker OR provider OR practitioner)) OR ((community) AND (worker* OR (health AND care AND provider*)) OR (health AND worker*) OR (healthcare AND worker*) OR (primary AND care provider*) OR (primary AND healthcare AND provider*) OR (health AND officer) OR (health AND promoter) OR (health AND volunteer*) OR (health AND assistant*) OR (physician AND assistant*) OR (medical AND assistant*) OR (clinical AND officer*) OR (medical AND technician*) OR paramedic* OR ((lay) AND (worker OR (health AND worker))) OR (health AND counsellor) OR counsellor OR ((lay OR health) AND counsellor)   1. **Intervention**   train* OR teach* OR educate OR upskill* OR instruct OR ((career OR staff OR profession* OR personal OR personnel) AND (development)) OR (capacity AND build*)   1. **Context**   (south* AND asia*) OR bangladesh* OR India* OR bhutan* OR nepal* OR pakistan* OR (sri lanka*) OR maldiv* OR afghan* OR Thai* or North Korea* OR myanmar OR Timor-Leste   1. **Mental health context**   anxi* OR depress* OR (mood AND disorder) OR (mental AND health) OR (mental AND disorder) OR (mental AND illness) or suicid* OR (psychiatric AND disorder) OR (psychological AND disorder*) or (psychological AND illness*) OR (psychological AND problem*) OR (psychiatric AND disorder*) OR (psychiatric AND illness*) OR (psychiatric AND problem*)  **Total (1+2+3+4)** | **328,358** **1,880,498** **113,551** **486,502**  **16** |
